# The progress of brazilian neuropsychology: From research to clinical practice

**DOI:** 10.1590/S1980-57642016DN10100013

**Published:** 2016

**Authors:** Emmy Uehara

**Affiliations:** 1 Professor, Department of Psychology, Federal Rural University of Rio de Janeiro, Rio de Janeiro RJ, Brazil

To present the new edition of **Neuropsicologia Hoje** is a task that makes me feel truly honored and grateful; this book was part of my training as a neuropsychologist and researcher. It was first published in 2004 with the aim of presenting neuropsychology to graduate students, updating professional skills and discussing new research findings. Thus, the book completed its 10th anniversary of the landmark first edition, being considered one of the pioneers to disclose the neuropsychology in Brazil and a reference work in Portuguese. Since its first publication, the Brazilian neuropsychology has grown and clearly shown a great progress, which is reflected by the expansion of the book. Now, it contains ten new chapters on topics, which are not presented in the first edition, including an entirely part of intervention. The book consists of 5 parts and 33 chapters covering a range of theoretical and practical aspects. The organization of the book follows the multidisciplinary integration model expressed by the renowned professionals from different fields, such psychologists, speech therapists, psychiatrists, and neurologists among others.

The first part of the book "General Aspects" presents a brief overview of the main conceptual frameworks that underpin our current understanding of brain structure and functioning. In the first chapter, "Morphofunctional Bases of the Nervous System", the biologist Alfred Sholl-Franco provides colorful illustrations that enrich even more the content and the description of the whole structure of the nervous system. Intelligence, processes of attention, executive functions, memory systems, language and emotional aspects are some of the main domains in cognitive and experimental neuropsychology, presented along the next seven chapters. The subsequent three chapters (9, 10 and 11) shows the importance of neuropsychology evaluation by bringing forth information about methodological approaches, clinical recommendations, data interpretations and *instruments adaptation process. In other words, the part I is an e* ssential content for the neuropsychologist formation.

Attention Deficit Hyperactivity Disorder (ADHD), Autism Spectrum Disorder (ASD) and Developmental Dyslexia are some of the disorders discussed in the second part of the book - "Childhood and Youth". José Salomão Schwartzman describes the clinical condition of Autism Disorder, emphasizing the importance of the early detection and intervention of autism and pervasive developmental disorders. In the next chapter, Paulo Mattos discusses about the request of neuropsychological assessment in different cases of ADHD suspect. The Speech therapists Simone Capellini and Renata Mousinho accounts for the conceptualization of the term Dyslexia and gives a classical profile of a student with a learning disability in reading. As well as Developmental Dyslexia, Dyscalculia is a disorder that affects negatively the school learning and adaptive behavior if untreated. In chapter 15, Haase, Júlio-Costa and Santos explains these difficulties in arithmetic skills and numerical competence by focusing on the Dehaene and Cohen's Triple-Code Model (1995). Lastly, Mauro Muskat, Mônica Miranda and Débora Muskat devoted to adolescent neuropsychology focus on the differences in brain processing information, structural neurobiology, neuroendocrine changes and sleep patterns that occur during the teen years. It is important to highlight that each chapter provides updated information according to the Diagnostic and Statistical Manual of Mental Disorders, fifth edition (APA, 2013). Therefore, I would recommend it for neuropsychologists, but also to all clinicians and professionals dealing with the development in childhood and adolescence.

In Part III, entitled "Adulthood", seven chapters explore some issues inherent to this stage of the development. Eliane Miotto starts describing acquired brain injury, especially the traumatic brain injury (TBI). She points to the main cognitive and behavioral sequelae that affect TBI survivors. Multiple sclerosis (MS) is an immune-mediated inflammatory disease that attacks myelinated axons in central nervous system. Andrade and colleagues detail many aspects about MS, such epidemiology, symptoms, psychiatry manifestations and neuropsychological assessment. As the authors stated: "*the research and clinical practice must go in parallel, so knowledge about MS can be enlarged* ". The 19th chapter is devoted to the neuropsychological assessment of epilepsy. The neuropsychologist Mäder-Joaquim provides a comprehensive information about the neuropsychological approach related to cognitive aspects of epilepsy. In the next chapter, Alessio, Damasceno, Ozelo and Cendes complement Mäder's narrative discussing the role of neuroimaging in memory evaluation in patients with refractory epilepsy of temporal and frontal lobes. The chapter 21 deals with a subject that is rarely discussed in neuropsychology, the HIV-associated neurocognitive disorders (HAND). Elisabete Konkiewitz and Flávia Heloísa Santos provide us a great opportunity to learn more about this disorder and clinical challenges related to it. The neuropsychology of bipolar disorder is discussed in chapter 22. The psychologists Malloy-Diniz, Lima and the psychiatry Neves explore dimensional characteristics of the neuropsychiatric of Bipolar disorder that contribute to their morbid manifestations such as suicidal behavior and functional disability. Lastly, Mograbi and Landeira-Fernandez offer the reader a thorough overview of the neuropsychological deficits in panic disorder. Thus, each chapter describes carefully the clinical condition as well as particular issues about those disorders and can be read separately without affecting the comprehension.

Population aging is a global phenomenon that has direct consequences on public health systems and in the worldwide economy (United Nations, 2013). With the increasing number of elderly in Brazil, further studies are needed in order to understand the cognitive processes involved in normal aging as well as in cognitive changes associated with common diseases of the elderly. Part IV "Senescence" offers a summary of the frequently diseases encountered in older adults, for example, the first chapter reviews a fatal neurodegenerative disease of the human motor system: the Amyotrophic Lateral Sclerosis (ALS). Berenguer-Pina & Santos describe the prevalence, etiology, clinical features, cognitive deficits and treatment. Sonia Brucki presents in chapter 25 an overview about the relationship between memory loss in aging and mild cognitive impairment (MCI). The next chapter, Behavioral Variant of Frontotemporal Dementia (bvFTD), Bahia, Takada e Nitrini explain this form of frontotemporal degeneration, characterized by behavioral disinhibition, apathy, loss of empathy, change in eating habits and executive dysfunction. Paulo Bertolucci describes the Alzheimer's disease (AD) as the most common form of dementia among older adults. One of the first signs is memory problems; however, they vary from person to person. At the end of the chapter, the author provides tips to maintain healthy brain. In the last chapter, Maia, Cardoso and Caramelli give an overview of Parkinson's disease (PD). It is a progressive disorder of the nervous system, which mainly affects movement, such as muscle rigidity, tremors and changes in speech and gait.

In the last decades of the XX century, there has been considerable interest in the technologies innovations in neuropsychology field. In this context, the use of technology takes place through different ways in research and practice exemplified in Part V - "Interventions". For example, emerging technologies as noninvasive brain stimulation strategies written by Boggio and colleagues and the brain training products in cognitive rehabilitation discussed in chapter 31 by Fabiana Ribeiro and Flávia Heloísa Santos. On the other hand, traditional methods and techniques are still used in clinical practice. Claudia Berlim presents some models of cognitive rehabilitation in childhood, which are also described by Monica Yassuda and Paula Brum, focusing on Mild Neurocognitive Disorder (MNC) in older adults. At the same time, Silvia Bolognani explains the 'Hypothesis Table', a new tool for neuropsychology intervention. These resources may be another option to enhance the intervention in the recovery and a non-pharmacological alternative to develop the autonomy and maintain the quality of life of patients and their families. Throughout the book, the value of evidence-based interventions that gives more reliability and accuracy regarding the therapeutic and preventive approaches used. 

Finally, the reviewed book provides an update review for beginners and for professionals of the current neuropsychology research and clinical practice. Furthermore, for all those who are interested in the topic of neuropsychological evaluation and intervention strategies related to lifespan development. I am sure the reader will find this book as useful in their clinical practice as interdisciplinary study for teaching and training. I hope you all have a great time reading it!
